# Extent of morbidity associated with schistosomiasis infection in Malawi: a review paper

**DOI:** 10.1186/s40249-015-0053-1

**Published:** 2015-05-04

**Authors:** Austin H N Mtethiwa, Gamba Nkwengulila, Jared Bakuza, Daniel Sikawa, Abigail Kazembe

**Affiliations:** Lilongwe University of Agriculture and Natural Resources (LUANAR), Bunda Campus, PO Box 219, Lilongwe, Malawi; Zoology Department, University of Dar es Salaam, College of Natural and Applied Science, PO Box 35064, Dar es Salaam, Tanzania; Biological Sciences Unit, Dar es Salaam University College of Education (DUCE), PO Box 2329, Dar es Salaam, Tanzania; University of Malawi, Kamuzu College of Nursing, Private Bag 1, Lilongwe, Malawi

**Keywords:** Schistosomiasis, Morbidity, Risk factors, Quantification, Burden, Extent

## Abstract

Data on the extent of the burden due to schistosomiasis is sparse in most Sub-Saharan African countries. However, this data is crucial for triggering medical attention. A review of extent of morbidity and determinants associated with schistosomiasis in Malawi was therefore conducted to quantify the infection in order to concretise the need for medical intervention. A systematic and traditional search strategy was used to find literature for the review, whilst exclusion and inclusion criteria were used to identify appropriate articles. Logistic regression curves of epidemiological model Y = (*a* + *bx*^*c*^)/(1 + *bx*^*c*^) and the recommendation that schistosomiasis prevalence can be used to estimate morbidity were employed to quantify morbidity at various infection stages. Morbidity was quantified as a direct proportion of the population and the respective national schistosomiasis prevalence. Findings showed that both S*. mansoni* and *S. haematobium* are present in Malawi with the latter highly prevalent (50%). Furthermore, out of the estimated population of 16,829 million, approximately 8.4 million have schistosomiasis, with about 4.4 million of these aged 18 years and below. The most frequent manifestation is Katayama syndrome, while ascites is the lowest, impacting about 3.0 million and 960 individuals, respectively. Localised studies on association of schistosomiasis infection to risk factors such as occupation, age and gender found odds ratio (OR) ranging from 1.29 to 5.37. Morbidity due to schistosomiasis is high in Malawi. It is therefore recommended that a more detailed study on the determinants of high schistosomiasis and re-evaluation of the current control measures be conducted if the current morbidity statistics are to be remarkably reduced.

**Electronic supplementary material:**

The online version of this article (doi:10.1186/s40249-015-0053-1) contains supplementary material, which is available to authorized users.

## Multilingual abstracts

Please see Additional file 1 for translations of the abstract into the six official working languages of the United Nations.

## Introduction

There are both urinary and intestinal schistosomiasis present in Malawi, but their prevalences vary widely in space and magnitude [1,2]. Despite the existence of the disease, officially harmonised morbidity, mortality, or associated mortality and morbidity data are not readily available. This paper is a review of the relevant studies conducted on schistosomiasis in Malawi with special focus on morbidity, prevalence and determinants (risk factors, and knowledge, attitude and practices [KAP]) of the disease.

Malawi is a country located in Southern Africa and occupies an area of approximately 119,000 km^2^. It has an estimated population of 16,829 million [3,4]. The country is bordered by Zambia in the west, Tanzania in the north and northeast, and Mozambique in the east, south and southwest. It is subdivided into three administrative regions: Southern, Central and Northern [3].

Schistosomiasis, also known as bilharziasis or bilharzia [5], is one of the chronic parasitic diseases caused by digenetic trematodes of the genus *Schistosoma*. Humans can acquire it through contact with cercariae-infested freshwaters. It is endemic in 78 countries and is caused by at least seven schistosome species which include *S. haematobium, S. mansoni, S. japonicum, S. intercalatum, S. malayensis, S. mekongi* and *S. sinensium* [5,6]. *Schistosoma haematobium* and *S. mansoni* are the two species most endemic in Sub-Saharan Africa causing urinary and intestinal schistosomiasis, respectively [7]. *Bulinus* and *Biomphalaria* species are the intermediate host snails for *S. haematobium* and *S. mansoni*, respectively [8].

The life cycle requires surface freshwater in which the parasite eggs from infected humans will hatch into miracidia. The miracidia penetrate an appropriate aquatic snail where they mature into cercariae. The cercariae then leaves the snail and penetrates the human skin and develops inside the body to maturity [9,10]. The matured schistosomes have separate sexes and the male body has a groove where the female is held for the rest of its life, releasing fertilised eggs [10]. Among the common signs, intestinal schistosomiasis causes fatigue, abdominal pain, diarrhoea and blood-stained stools, while urinary schistosomiasis causes dysuria, frequent urination and terminal haematuria [11]. Schistosomiasis can be diagnosed microscopically by examining schistosome eggs in the stool (*S. mansoni*) or urine (*S. haematobium*) [12]. In addition, urinary schistosomiasis can be detected by the presence of blood in the urine and intestinal schistosomiasis by blood in the stool [9]. A number of the symptoms and signs of schistosomiasis infection are common to other diseases, and as such, it is not easy to isolate and estimate the extent to which schistosomiasis can cause morbidity in the human population [13]. It is with this background that this review was conducted to quantify the morbidity due to schistosomiasis in Malawi.

## Review

**Figure 1:**
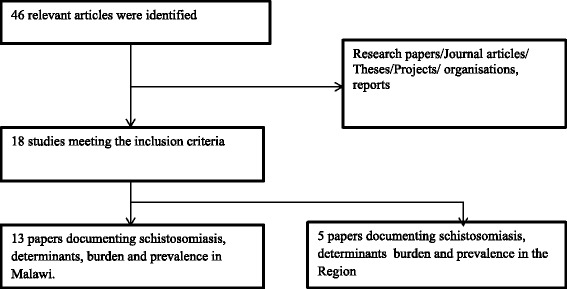
Decision tree showing inclusion and exclusion criteria for identifying of studies.

An integrated literature search, comprising traditional and systematic literature search and review approaches, was used. The retrieved literature was screened and synthesised according to a set of predetermined criteria. The systematic literature search was conducted using PubMed, Global Health, HINARI and World Health Organization (WHO) databases and relevant journals, whilst a traditional literature search collected information from different institutions, Google, bibliographies of identified papers and grey literature. Institutions that were consulted in Malawi included the Ministry of Health (MoH), the Bilharzia Control Programme office, College of Medicine Library and various individuals. A decision tree for inclusion and exclusion of articles was used, as described in [14] and outlined in Figure 1.

### Screening of relevant articles

Inclusion and exclusion criteria were used to select articles to include in the review. These were based on dates, languages, titles and content. The search included articles written in English between 1970 and 2014. Studies conducted either in Malawi or within the Malawi region, or with international coverage but that had relevant information to this review, were selected. Forty-six articles were identified and of these, 18 met the inclusion criteria. To identify relevant studies, text strings ‘*Bulinus/Biomphalaria* or Malawi’, ‘*S. haematobium* or Malawi’, ‘*S. mansoni* or Malawi’, ‘burden or Malawi’, ‘prevalence’, ‘Malawi’ or ‘schistosom*’ and ‘urinary’ or ‘Malawi’ were used.

Literature was screened in two phases. The first phase was based on titles and abstracts of the retrieved articles, whilst the second stage was applied to studies that passed the first phase and focused on content, methodology and outcomes. Full-text papers that passed the second stage were reviewed and interpreted against the objectives of this review. The final list of the identified studies/articles on schistosomiasis is outlined in Table 1.

**Table 1:** **List of articles that met the inclusion criteria for the review**

**SN**	**Topic**	**Year**	**Region**	**Sample size**	**Burden (DALYs)**	**Morbidity**	**Mortality**	**Prevalence**
A1	Schistosomiasis Morbidity and management cases in Africa	2003	Africa (Review paper)	--	1.3	--	--	0-89
A2	Global burden of diseases due to schistosomiasis	2003	World (Review paper	--	1.7	220,000	--	--
A3	National survey on the prevalence of schistosomiasis and soil helminths in Malawi	2004	Malawi	1,664	--	--	--	6.9
A4	Prevalence, distribution and risk factors of S. haematobium infection in schoolchildren in Blantyre Malawi	2009	Malawi, Blantyre	1,139	--	--	--	0-46
A5	Schistosomiasis and water resources development: systematic review, meta-analysis, and estimates of people at risk	2006	Africa (review paper)	--	4.7	--	--	0-67
A7	Schistosomiasis Control Programme - Community Health Surveillance Unit (1997–2001): Lakeshore Schistosomiasis Control Project.	2001	Malawi	--	--	--	--	40-50
A8	Analysis of Schistosomiasis haematobium Infection Prevalence and Intensity in Chikhwawa, Malawi: An Application of a Two Part Model.	2013	Malawi, Chikhwawa	1642	--	--	--	14.3
A9	2008 Population and housing census, Minister of Economic Planning and Development, National Statistical Office, Malawi.	2008	Malawi	~14,000,000	--	--	--	--
A10	The schistosomiasis intermediate host *Bullinus nyassanus* is a preferred food for cichlid fish-*Tramacranus placodon* at Cape Maclear, Lake Malawi.	2006	Malawi, Mangochi	--	--	--	--	
A11	National Survey to find out difficulties people face in taking regular antischistosomal drugs in Malawi.	2003	Malawi	--	--	--	--	--
A12	The burden of disease in Malawi.	2006	Malawi	--	--	--	--	--
A13	Measuring the global burden of disease	2013	187 countries (A review paper)	--	1.7-4.7	--	--	0-67
A14	Schistosomiasis in Lake Malawi	1994	Malawi	--	--	--	--	32
A15	Schistosomiasis in Lake Malawi and the Potential Use of Indigenous Fish for Biological Control	2012	Malawi	487	--	--	--	43
A16	Schistosomiasis in Lake Malawi Villages	2011	Malawi	487	--	--	--	94
A17	Bulinus nyassanus is an intermediate host for Schistosoma haematobium in Lake Malawi	2001	Malawi	--	--	--	--	--
A18	Sentinel surveillance of Lymphatic filariasis, Schistosomiasis, Soil transmitted helminths and Malaria in rural southern Malawi	2010	Malawi	1,903	--	--	--	94

### Occurrence and prevalence

Schistosomiasis has been endemic in Malawi for decades [15] and has been recognised for more than 80 years [16]. Current statistics show high infection rates in children aged between five and 15 years, with prevalences of 90–100% in highly endemic areas. This is due to children’s higher exposure and dependence on the infected water bodies, as well as the fact that children are less resistant than adults because of the children’s low exposure to worm antigens [17-19]. Currently in Malawi, it is estimated that the national prevalence for schistosomiasis is between 40% and 50% [17], as reported in [18,20]. However, other findings show that 80% of the Malawian population is at risk of infection with most of those infected living in rural areas [17]. Research has shown that there has been a sharp increase in prevalence from the mid-1980 along the lakeshore areas of Lake Malawi [21]. Furthermore, Chitipa, Karonga, Nkhata Bay, Kasungu, Nkhotakota, Salima, Mangochi, Machinga, Zomba, Chikwawa and Nsanje are districts that have recorded high prevalences [22]. High prevalence rates are presumably enhanced by either their proximity to large water bodies (lakes and rivers), rice farming and sources of water for domestic use [14]. Consequently, most of the districts in Malawi do not have local estimates [2].

Literature further revealed that in Malawi, *S. haematobium* is highly prevalent and more predominant in the southern region, while *S. mansoni* is more prevalent in the central and the northern regions [15]. This can be due to variations in the abundance and distribution of the *Biomphalaria* and *Bulinus* snail, which is an effect of selective introductions and climatic factors within different ecological zones [23]. Multiple/mixed infections with *S. haematobium* and *S. mansoni*, hookworms and *Ascaris* have been reported in a number of studies, with a prevalence of about 12% [1,18]. However, prevalence in communal water reservoirs has not been adequately documented.

### The burden of schistosomiasis in Malawi

Recent global findings rank schistosomiasis as one the 25 leading diseases [24]. In Malawi, schistosomiasis is among the top 20 diseases causing high outpatient attendances at health facilities [22]. Beside this, the disease ranks second only to malaria among the parasitic diseases in terms of the number of people infected and at risk in endemic areas [22,25]. It leads to a loss of 1.7–4.5 million disability-adjusted life years (DALYs) in the world [6]. Consequently, schistosomiasis contributes approximately 0.1% and 0.4% to the Sub-Saharan Africa and global disease burden, respectively [13]. However, considering the large number of people that are infected, the small estimated contribution by schistosomiasis to the disease burden may not be a true reflection, and may be masked by the immerse burden of HIV/AIDS, malaria, childhood diseases, diarrhoeal diseases and tuberculosis [13]. Furthermore, isolating morbidity caused by schistosomiasis from that caused by other diseases is complicated [2]. One method to achieve this is to identify all diseases that cause a certain common morbidity in a population and treat each separately. The morbidity rate that remains can then be attributed to schistosomiasis. A complication again arises as some of the drugs are active against several morbidities and/or diseases. The burden of schistosomiasis has not been determined in Malawi using DALYs.

Based on the current population of 16 million and a schistosomiasis prevalence of 50% [17,20], it can be estimated that about eight million people are infected in Malawi. It further translates that about 7.2 million of the infected population are found in rural areas since about 90% of Malawians live in these areas [3]. Approximately 4.2 million (52%) of the infected population is aged 18 years and below because 52% of the Malawi population is within that age range [3]. The implication of this is that the country will end up with a sick, unproductive generation if schistosomiasis patients are not treated on time. Amongst school-age children, prevalence is higher in males than in females due to behavioural and occupational variabilities that lead to differences in the frequency of visits to water bodies. Studies have shown that those who frequent water bodies for various activities are at a higher risk of contracting the disease than their non-visiting counterparts [2].

### Morbidity

Morbidity is a non fatal outcome while mortality is a fatal outcome of an ailment [4,13,26]. Schistosomiasis morbidity exhibits in various stages, as shown in Table 2.

**Table 2:** **Schistosomiasis morbidity at various infection stages**

**Stage**	**Pathology**	**Morbidity due to** ***S. mansoni***	**Morbidity due to** ***S. haematobium***
Invasion		Cercarial dermatitis	Cercarial dermatitis
Acute	As a result of antigens and metabolites excreted with egg production	Katayama fever, weakness, weight loss, headache, anorexia, nausea, vomiting, diarrhoea, dry cough, hepatosplenomegaly, bloody diarrhoea, urticaria, periorbital oedema, bronchospasm	Katayama fever, chills, weakness, weight loss, headache, haematuria, pain on micturition, urinary dribbling and incontinence,
**Early**	Colonic focal fibrosis and granulomatous inflammation	(Bloody) diarrhoea, blood in stool, abdominal pain	haematuria
**Early and late**		Anaemia	
**Late**	Portal hypertension	Hepatosplenomegaly ascites, oedema, oesophageal varices haematemesis, liver failure, corpulmonale	Bladder cancer, obstructive uropathy, hydronephrosis, renal parychyma impared, kidney disfunction
**Subtle**	Ectopic lesions (CNS)	Convulsions, paralysis, reduction of growth, impaired cognitive development, reduced physical fitness	Uropathy, kidney failure
**Mortality**	Portal hypertension	Liver failure, corpulmonale Haematemesis	Kidney failure, ascetic renal failure, bladder cancer, kidney necrosis

Source: [9,26,40,41].

Using a relationship (regression) model^a^: Y = (*a* + *bx*^*c*^)/(1 + *bx*^*c*^), which stipulates that prevalence of a specific morbidity is a function of prevalence of the national schistosomiasis infection [26], and a recommendation that ‘prevalence of an infection is the only readily available epidemiological parameter that can be used to estimate morbidity’ [13], the prevalence of a specific morbidity at each stage of schistosomiasis infection can be estimated. The proportion of individuals with a specific morbidity is estimated from the community or national prevalence rates, as outlined in Table 3.

**Table 3:** **Estimates of morbidity ratios at respective schistosomiasis prevalence**

**Morbidity/infection**	**0.15**	**0.50**	**0.85**
***S. mansoni***			
Katayama	0.012	0.002	0.091
Diarrhoea	0.0001	0.001	0.072
Blood stool	0.001	0.02	0.24
Hepatomegaly (MSL)	0.01	0.07	0.14
Hepatomegaly (MCL)	0.01	0.06	0.12
Splenomegaly	0.011	0.047	0.089
Haematemesis ever	0.002	0.006	0.011
Ascites	0.0004	0.0012	0.0021
***S. haematobium***			
Katayama	0.062	0.232	0.387
Haematuria	0.031	0.197	0.349
Incontinence	0.011	0.047	0.089
Bladder cancer	0.01	0.06	0.12
Kidney failure	0.01	0.07	0.14

Source: [13,24,26].

Based on the national prevalence of 50% and 10% for *S. haematobium* and S*. mansoni*, respectively [2,20], and subsequent proportions outlined in Table 3, the number of individuals with specific schistosomiasis morbidity is estimated and presented in Table 4. The estimates show that in Malawi, there are about 2.5 million people inflicted with Katayama and about two million with haematuria. However, Van der Werf [26] estimated that 20% (3.2 million) of the Malawi population experience schistosomiasis-related dysuria and haematuria. The estimate by this study is lower than that estimated by Van der Werf [26]. The difference could be as results of disease intervention over the 10 years after the estimate. Besides this, considering that 52% of Malawians are below 18 years of age, it can be said that more that half of those inflicted by these ailments are children. Mortality models are not available hence estimates of mortality caused by schistosomiasis could not be calculated.

**Table 4:** **Number of individuals with various** ***S. haematobium*** **and** ***S. mansoni*** morbidity in Malawi at respective prevalence*

**Morbidity/infection**	**0.15**	**0.50**	**0.85**
***S. haematobium***			
Katayama		2,256,800	
Haematuria		1,654,800	
Incontinence		394,800	
Bladder cancer		504,000	
Kidney failure		588,000	
***S. mansoni***			
Katayama	126,219		
Diarrhoea	2,520		
Blood stool	2,520		
Hepatomegaly (MSL)	27,768		
Hepatomegaly (MCL)	27,768		
Splenomegaly	27,768		
Haematemesis ever	5,048		
Ascites	1,009		

*Morbidity = morbidity ratio (at respective national schistosomiasis prevalence) x number of individual infected by schistosomiasis.

### Control strategies for schistosomiasis

A number of measures are employed aimed at preventing new infections, usually by interrupting the parasite’s life cycle [27]. This has been done by using methods that either eliminate the intermediate host or the parasite from the definitive host, or prevent infection of the definitive host or of the intermediate host [9,21,22,28]. Apart from the chemotherapy intervention, an integrated approach is employed where several control measures are combined. An essential component of the integrated control strategy, as outlines by the WHO, is the provision of ultimate health care to patients in hospitals [29].

#### Elimination of intermediate host snails

In Malawi, there is sketchy information on the biological and chemical approaches for the control of the intermediate host snail. Biologically, the *Trematocranus placodon* fish of the family *Cichlidae,* which feeds on *Bulinus* and *Biomphalaria* snails species, has been studied and used to control snails on an experimental basis, however, results have not been remarkable [21,30]. Decline in the *T. placodon* population due to heavy artisanal fishing and its possible preference for soft foods to snails have been indicated as the setbacks for the success of the intervention [30]. Unless research on this is intensified, its application in the field in Malawi remains uncertain.

Use of molluscicides in Malawi has been largely on individual farms, especially fishponds, with no formal documentation on its implementation, coverage and success. Whilst this approach may be reliable, it has portrayed a number of major shortfalls among which are severe toxicity non-target soft bodied aquatic organisms [31]. Niclosamide is the only commercially available molluscicides recommended by the WHO for large-scale use in schistosomiasis control programmes [27,32].

#### Chemotherapy

In Malawi, 60% of the health services are provided by the Ministry of Health and Population, 37% by the Christian Health Association of Malawi (CHAM), 1% by the Ministry of Local Government and 2% by other providers [3,22]. Chemotherapeutically, praziquantel has been used for years to treat and control schistosomiasis in Malawi. However, studies have shown that 30% of the population cannot access the drug [22]. This is because the drug is either not available at government health units, is expensive in the private pharmacies, or that long distances discourage and preclude people from getting to the government health units where the drug is provided [22,30]. Beside this, like in other countries, free drugs are mostly available to school children only [22,33]. Despite this, the Malawi National Schistosomiasis Control Programme does not have well-documented evidence of when and where the universal drug treatment has been offered [1]. One of the problems with this approach is re-infection that can happen after visiting the cercariae-infested waters after treatment, a probable problem in the country.

### Social determinants of schistosomiasis infection

The community’s knowledge, attitudes and practices (KAP) and risk factors influence acquisition, transmission and thus persistence of schistosomiasis in the community [34]. As such, a good understanding of local KAP is central to the development of effective control measures that would thwart further transmission [15]. Communities, especially children, lack understanding on the transmission of schistosomiasis [15,33]. In Tanzania, for instance, the prevalence of the disease remained as high as 62%, although 82% of the children were treated for it [35]. This may be attributed to a lack of knowledge on the transmission of schistosomiasis and unchanged behaviour in many of the school children [35,36].

Studies have unveiled that communities perceive schistosomiasis as a normal physiological development in growing children, a disease that recurs however you treat it, a disease for males, a sexually transmitted disease and as a disease better treated with herbs [34,35,37]. In light of these attitudes, it is unlikely that patients will seek treatment from hospitals [33]. In essence, KAP on schistosomiasis has not been adequately studied in Malawi.

#### Risk factors

Localised studies in Malawi identified occupation, age, education, gender, socioeconomic status, proximity and frequency of visits to water bodies or sources of water as common risk factors [2,18]. Furthermore, studies found a strong association of prevalence and risk factors, with an OR ranging from 1.72 to 5.39 [2]. These findings mean that these factors would determine the level of acquisition of schistosomiasis in Malawi.

#### Snail intermediate hosts

The distribution of schistosomiasis is determined, to a larger extent, by the presence or absence of snail intermediate hosts [23]. In Malawi, *Biomphalaria* and *Bulinus* have been identified, and at least studied, in the Lake Malawi ecosystems [38]. *Bulinus nyassanus*, one of the intermediate host for *S. haematobium* [39] is endemic to Lake Malawi and is found on open sandy areas without macrophytes, usually buried 2–3 cm into the gravel. Another snail that is an intermediate host for *S. haematobium* and is most common among aquatic plants is *B. globosus.* Although it is uncommon in Lake Malawi, it has been reported in several sites in the lake, especially near inflowing streams [39]. However, there is scanty information on the distribution, seasonality and infectivity of host snails in the communal water reservoirs.

## Conclusion

This review has unveiled alarming schistosomiasis morbidity statistics in Malawi amidst years of chemotherapeutical intervention. It has further revealed that risk factors, and knowledge, attitude and practices on schistosomiasis have not been adequately explored. It is expected that more information about the disease in the country will be disclosed as more studies are being conducted. Re-evaluation of the current control measures and implementation of integrated targeted and effective schistosomiasis control measures are recommended if the current morbidity statistics are to be remarkably reduced.

### Study limitations

Whilst every effort was made to gather all the relevant documents and information, a number of grey literatures on KAP for the study, including those conducted by the Danish Bilharzia Laboratory around the 1990s, could not be accessed. This is because the literature is not readily available in the public domain. However, since this review mostly focused on the quantification of morbidity and the rest of the information is only supportive, the absence of this literature would not significantly change the outcome of this review.

## Endnote

^a^*Y* denotes specific morbidity; *x* denotes schistosomiasis infection; *a* denotes prevalence due to other diseases; *b* and *c* denote degree of association where *b* = (*c*–1)/(c + 1)*.*

## Additional file

Additional file 1:
**Multilingual abstracts in the six official working languages of the United Nations.**
