# Supplementary material for: Extent of morbidity associated with schistosomiasis infection in Malawi: a review paper
Source: Infect Dis Poverty. 2015 May 4;4:25. doi: 10.1186/s40249-015-0053-1 (PMC4423108; doi:10.1186/s40249-015-0053-1)
Supplement: Additional file 1: — Multilingual abstracts in the six official working languages of the United Nations. [file 40249_2015_53_MOESM1_ESM.pdf]

Translation of the abstract into the six official working languages of the United Nations

**مدى انتشار الحالة المرضية المصاحبة لعدوى البلهارسيا في ملاوي: ورقة مراجعة**

أوستن إتش. إن متيثوا، جامبا نيكوينجليلا ، جيرد باكوزا، دانيال سيكاوا و أبيجال كازيمبي

**نبذة موجزة**
تعتبر البيانات المتعلقة بمدى المسؤولية المطلوبة تجاه مرض البلهارسيا قليلة وغير منسقة في معظم دول جنوب الصحراء الإفريقية؛ غير أن هذه المعلومات تعد ضرورية وهامة لجذب الانتباه الطبي. ومن هنا، قد تم القيام بمراجعة مدى انتشار المرض والعوامل المحددة المصاحبة لمرض البلهارسيا في ملاوي، وذلك من أجل تحديد حجم العدوى بغية إثبات ضرورة التدخل الطبي هناك. هذا وقد تم استخدام استراتيجية بحث منسقة ومعروفة للتوصل إلى تاريخ المراجعة، بالإضافة إلى استخدام معايير الإدراج والاستبعاد لتحديد العناصر الملائمة؛ كما تم استخدام منحنيات التراجع اللوجستي للنموذج الخاص بعلم الأوبئة $Y=(a+bx^c)/(1+bx^c)$، والتوصية بأن انتشار مرض البلهارسيا يمكن أن يتم استخدامه لتقدير مدى انتشار المرض وكذلك لتحديد حجم معدل انتشار المرض في مراحل العدوى المختلفة. هذا وقد تم حساب مدى انتشار المرض بين نسبة مباشرة من السكان، وكذلك انتشار العدوى بالبلهارسيا على المستوى المحلي المعني. وقد أظهرت النتائج أن كلاً من إس. مانسوني و إس. هيماتوبيوم موجودين في ملاوي حيث كان الأخير منتشر بنسبة مرتفعة هناك (50%). وعلاوة على ذلك، فإن هناك قرابة 8.4 مليون شخص- من إجمالي عدد السكان المقدر بحوالي 16.829 نسمة- يعانون من الإصابة بعدوى البلهارسيا، في حين يبلغ عدد المصابين بهذه العدوى في سن 18 عاما وما دونه قرابة 4.4 مليون نسمة. هذا ويعد متلازم كاتاياما هو المظهر الأكثر انتشاراً، في حين يعد الاستسقاء هو المظهر الأدنى، والذي يؤثر في قرابة 3.0 مليون و 960 شخص على التوالي. وتظهر الدراسات المحلية حول تلازم عدوى البلهارسيا لعوامل المخاطر مثل المهنة، السن والنوع معدلات غريبة تتراوح بين 1.29 إلى 5.37. يعد انتشار المرض كنتيجة لعدوى البلهارسيا مرتفعا في ملاوي. ومن ثم فإنه يوصى بأن يتم إجراء دراسة أكثر تفصيلا حول العوامل المحددة للانتشار الواسع لعدوى البلهارسيا وإعادة تقييم معايير وضوابط التحكم الحالية، إذا كانت هناك رغبة في تقليل المعدلات الإحصائية لانتشار المرض في الوضع الحالي بشكل ملحوظ.

Translated from English version into Arabic by Badran Hamed, through

TRANSLATORS
WITHOUT BORDERS

**马拉维的血吸虫病感染相关发病率回顾**

Austin H.N Mtethiwa, Gamba Nkwengulila, Jared Bakuza, Daniel Sikawa and Abigail Kazembe

**摘要：**
在很多撒哈拉以南的非洲国家，关于血吸虫病负担的资料不多。但是，这些资料对引起医疗关注是至关重要的。因此，为使医疗干预需求具体化，有必要总结马拉维的血吸虫病感染相关病情资料，从而来量化感染情况。本文采取传统的系统搜索方法来检索文献，使用排除和纳入标准来确定符合要求的文章。本文还采用流行病学模型“$Y=(a+bx^c)/(1+bx^c)$”Logistic 回归曲线和以血吸虫病患病率估算发病率的建议来量化不同感染阶段的发病率。发病率直接量化为总人口的比例以及各个国家的血吸虫病的流行程度。结果表明，马拉维有曼氏血吸虫和埃及血吸虫，后者的发病率非常高（50%）。此外，估计 16 829 000 人中，约有 840 万人患有血吸虫病，其中 18 岁及以下有 440 万。感染主要表现为片山综合症，偶见腹水，分别困扰 300 万人和 960 人。血吸虫病感染的危险因素，如职业、年龄和性别，其比值比（OR）的范围为 1.29-5.37。在马拉维，血吸虫病引起的发病率很高。因此，如果近期发病率大幅降低，建议深入研究血吸虫病严重流行的决定因素，并重新评估现行的防控措施。

Translated from English version into Chinese by Chen Jin, edited by Yin Jian-hai, through

CDC
CHINESE CENTER FOR DISEASE CONTROL AND PREVENTION
NATIONAL INSTITUTE OF PARASITIC DISEASES

**Rapport sur la charge de morbidité liée à la schistosomiase au Malawi**

Austin H.N Mtethiwa, Gamba Nkwengulila, Jared Bakuza, Daniel Sikawa et Abigail Kazembe

**EXTRAIT**
Les données portant sur le poids de la schistosomiase dans la plupart des pays d’Afrique sub-saharienne sont rares. Ces données sont pourtant cruciales pour déclencher une attention médicale. Une étude de la charge de morbidité liée à la schistosomiase et de ses facteurs déterminants au Malawi a été menée pour quantifier l’infection et concrétiser les besoins en intervention médicale. Une stratégie de recherche de traditionnelle et systématique a été adoptée pour

rechercher la documentation nécessaire à l'établissement de ce rapport, avec un recours des critères d'inclusion et d'exclusion pour identifier les articles appropriés. Des courbes de régression logistique du modèle épidémiologique $Y=(a+bx^c)/(1+bx^c)$ et la recommandation selon laquelle la prédominance de la schistosomiase peut servir à estimer la morbidité ont été utilisées pour quantifier la morbidité aux différents niveaux d'infection. La morbidité a été quantifiée en proportion directe de population et suivant la prédominance nationale de schistosomiase correspondante. Les résultats ont montré que *S. mansoni* et *S. haematobium* sont présents au Malawi, avec une prédominance nette du second (50%). De plus, sur une population totale estimée à 16 829 millions, environ 8,4 millions souffrent de schistosomiase, dont 4,4 millions âgés de 18 ans ou moins. La manifestation la plus fréquente est la maladie de Katayama, tandis que les ascites sont la moins fréquente. Leur impact est respectivement de 3 millions contre 960 personnes. Des études ciblées évaluant le lien entre la schistosomiase et certains facteurs de risques tels que la profession, l'âge, et le genre ont eu pour résultat des risques relatifs (*odds ratio*) allant de 1,29 à 5,37. La morbidité liée à la schistosomiase est élevée au Malawi. Il est donc recommander de mener une étude plus détaillée sur les déterminants de la schistosomiase aiguë et de réévaluer les mesures de contrôle actuelles afin de faire baisser les statistiques actuelles de morbidité.

Translated from English version into French by Fabienne Perrin, through

**Уровень заболеваемости инфекцией шистосомоза в Малави: обзорная статья**

Остин Мтетива, Гамба Нкенгулила, Джаред Бакуза, Дэниэл Сикава и Эбигейль Казембе

**Резюме**

Данные об уровне заболеваемости шистосомозом в большинстве африканских стран к югу от Сахары очень скудны. Тем не менее, эти данные необходимы для оказания медицинского обслуживания. Нами было проведено обзорное исследование уровня заболеваемости и детерминантов, связанных с шистосомозом в Малави, с целью дать количественную оценку этой инфекции и конкретизировать необходимость медицинского вмешательства. Мы использовали систематичный и традиционный подход к поиску литературы по теме и разработали критерии включения и исключения для того, чтобы определить подходящие статьи. Мы использовали кривые логистической регрессии эпидемиологической модели $Y=(a+bx^c)/(1+bx^c)$ и рекомендации о том, как применять коэффициент распространенности шистосомоза для оценки заболеваемости, с целью количественно измерить заболеваемость на разных стадиях инфицирования. Мы выразили заболеваемость прямо пропорциональной населению и соответственному национальному коэффициенту распространенности шистосомоза. Исследование показало, что оба паразита *S. mansoni* и *S. haematobium* присутствуют в Малави, и у последнего - высокая распространенность (50%). Более того, из общего населения в 16,8 миллиона 8,4 миллиона больны шистосомозом, из них 4,4 миллиона - в возрасте 18 лет и младше. Самое распространенное проявление болезни – синдром Катаямы, самое редкое – асцит, поражая около 3 миллионов и 960 человек соответственно. Локализированные исследования на предмет корреляции между инфекцией шистосомоза и такими факторами риска, как профессия, возраст и пол выявили отношение шансов (ОШ) в диапазоне от 1,29 до 5,37. В Малави наблюдается высокая заболеваемость шистосомозом. Таким образом, с целью существенного снижения актуального уровня заболеваемости рекомендуется проведение более глубокого исследования детерминантов высокого уровня шистосомоза и переоценка актуальных мер борьбы с этим заболеванием.

Translated from English version into Russian by Nurangiz Khodzharova, through

**Grado de morbilidad asociada con la infección de la esquistosomiasis en Malawi: resumen de los avances**

Austin H.N Mtethiwa, Gamba Nkwengulila, Jared Bakuza, Daniel Sikawa and Abigail Kazembe

RESUMEN

Los datos sobre sobre el alcance de la carga del parásito de la esquistosomiasis son escasos en la mayor parte de los países de África Subsahariana. Sin embargo, esta información es crucial en la activación de una atención médica. Es por eso que se realizó una revisión sobre el grado de morbilidad y los determinantes asociados con la esquistosomiasis en Malawi para así cuantificar la infección y concretar la necesidad de intervención médica. Se utilizó una estrategia de búsqueda sistemática y tradicional para recopilar la literatura y revisarla, fueron empleados criterios de exclusión e inclusión para identificar los artículos apropiados. Las curvas del modelo de regresión logística de epidemiología Y(abxc)(1bxc) y la afirmación sobre una prevalencia de la esquistosomiasis fueron tomadas en cuenta para cuantificar la morbilidad en las distintas etapas de la infección. La Morbilidad se cuantificó como una proporción directa de la población y como una prevalencia de la esquistosomiasis en las regiones respectivas. Los resultados demostraron que tanto el Schistosoma Mansoni y Schistosoma Haematobium están presentes en Malawi, este último prevalece en un (50 %). Además de la población estimada de 16829 millones de personas, aproximadamente 8,4 millones tienen esquistosomiasis, y unos 4,4 millones entre estos tienen 18 años de edad o son menores. La manifestación más frecuente es el síndrome de Katayama, mientras que la ascitis es más baja, y afecta a unos 3 millones y 960 mil personas. Los estudios limitados a la relación entre la esquistosomiasis y los factores de riesgos asociados a la ocupación, la edad y el género mostraron odds ratio que oscilan entre 1.29 y 5,37. Por lo tanto, se recomienda realizarse un estudio más detallado sobre los determinantes de una esquistosomiasis alta y se haga una nueva evaluación de las medidas actuales de control verificando si las estadísticas de morbilidad actuales van a reducirse notablemente.

Translated from English version into Spanish by Karina Atencio, through

TRANSLATORS
WITHOUT BORDERS
